# Evaluating the Health Economic Impacts of Baloxavir Marboxil and Oseltamivir for the Treatment of Influenza in Adult Outpatients in Hong Kong: A Cost‐Effectiveness Analysis

**DOI:** 10.1111/irv.70243

**Published:** 2026-03-05

**Authors:** Ruohan Chen, Zengyang Shao, Kaiming Bi, Benjamin John Cowling, Zhanwei Du

**Affiliations:** ^1^ WHO Collaborating Center for Infectious Disease Epidemiology and Control, School of Public Health, LKS Faculty of Medicine The University of Hong Kong Pokfulam Hong Kong Special Administrative Region China; ^2^ Laboratory of Data Discovery for Health Limited Hong Kong Science and Technology Park New Territories Hong Kong Special Administrative Region China; ^3^ School of Public Health The University of Texas Health Science Center at Houston Houston Texas USA

**Keywords:** antiviral, baloxavir marboxil, cost‐effectiveness analysis, high‐risk adults, Hong Kong, influenza, oseltamivir, resistance

## Abstract

**Background:**

Baloxavir marboxil, an antiviral used only in private hospitals in Hong Kong since February 2019 for treating influenza, is recognized for its efficacy against strains resistant to standard antiviral agents, providing an alternative to neuraminidase inhibitors such as oseltamivir.

**Methods:**

This study compares the cost‐effectiveness of baloxavir and oseltamivir for influenza treatment in otherwise healthy (OwH) and high‐risk (HR) adult outpatients in Hong Kong from the healthcare‐payer's perspective. A decision tree was utilized to study the progression of influenza and estimate associated costs and quality‐adjusted life‐years (QALYs) over 14 days for each antiviral.

**Findings:**

In the general population, the incremental cost‐effectiveness ratio (ICER) for baloxavir is $89,921 per QALY. Compared to oseltamivir, baloxavir shows a QALY gain with an incremental cost of $13,626 per QALY, below the $152,667 willingness‐to‐pay (WTP) threshold. At this WTP level, there is a 92% chance the additional cost of baloxavir is accepted. As oseltamivir resistance rises, its economic benefit decreases, while baloxavir's improves. In the OwH population, baloxavir's ICER is $145,316 compared to baseline and $115,811 compared to oseltamivir, with a 49% probability of baloxavir being cost‐effective. In the HR population, baloxavir dominates, with ICERs of $36,163 and $‐15,072, and a 99% probability of cost‐effectiveness.

**Conclusion:**

As a result, baloxavir could potentially be considered as the influenza agent of choice over oseltamivir in Hong Kong, despite a higher acquisition cost. Therefore, we recommended that integrating baloxavir into the Hospital Authority Drug Formulary in Hong Kong could enhance healthcare resource allocation and improve patient outcomes.

## 1 Introduction

Influenza is an acute respiratory infection caused by influenza viruses that circulate globally [1]. The severity of influenza virus infections can vary widely, ranging from mild upper respiratory symptoms to severe pneumonia caused directly by the virus or secondary bacterial infections of the lower respiratory tract [2]. In some cases, influenza leads to significant illness, hospitalization, and even death. According to the World Health Organization (WHO), annual influenza epidemics are responsible for approximately 1 billion infections, 3–5 million cases of severe illness and 300,000–500,000 deaths worldwide [2]. This underscores the substantial global and year‐round disease burden posed by influenza. The virus is characterized by annual seasonal epidemics, with peaks typically occurring during winter in temperate climates. For instance, during the 2023–2024 winter influenza season in Hong Kong, which began in early January, the estimated influenza‐like illness (ILI) positive rate reached 44.67 per 10,000 people, with a public hospital admission rate of 0.55 per 10,000 people. This season saw 1175 reported severe cases and 799 influenza‐related deaths as of July 2024 [3].

Since 2004, the Hong Kong government has implemented the Seasonal Influenza Vaccines (SIV) Programme to provide free vaccinations to high‐risk groups, with the aim of mitigating the public health impact of influenza [4]. In the 2023–2024 program, overall vaccine coverage rate reached 52.9%, reflecting a 20% increase compared with the previous year [5, 6]. However, healthy persons aged 18 to 50 are not included in the priority group for SIV and must pay for the vaccine out‐of‐pocket, which deters individuals from seeking vaccinations [7]. Additionally, newly emerging influenza subtypes can render existing vaccines less effective, and the time required to develop new vaccines for these strains may limit their effectiveness [8]. The 2023–2024 winter influenza season in Hong Kong has been notably extended, with the Centre for Health Protection attributing this prolonged duration to changes in circulating influenza virus strains [9]. Given this, antivirals play a crucial role as a complementary measure to vaccination in reducing the medical and economic burden of influenza [10].

Oseltamivir, a neuraminidase inhibitor available in capsules or oral suspension (Tamiflu), is FDA‐approved for treating uncomplicated acute influenza in patients [11]. It is recognized by the Hong Kong Special Administrative Region (HKSAR) Government as a recommended oral antiviral. As a part of its Influenza Pandemic Plan, the government has stockpiled approximately 11 million doses of antivirals, with oseltamivir accounting for about 90% of the supply, including both capsules and oral suspension, as of April 2023 [12]. However, resistance to oseltamivir has been observed during the treatment of seasonal influenza since early 2024 [13]. Most persons infected with oseltamivir‐resistant seasonal Influenza A (H1N1) virus strains had neither received prior oseltamivir treatment nor been exposed to individuals undergoing oseltamivir treatment or chemoprophylaxis [14]. To address this challenge, researchers are investigating novel compounds with different mechanisms of action to overcome specific drug resistance [15].

Baloxavir marboxil (Xofluza), developed by Roche and Shionogi, is an oral cap‐dependent endonuclease inhibitor that prevents influenza virus replication by inhibiting the initiation of mRNA synthesis [16]. In the CAPSTONE 1 trial, baloxavir demonstrated superior efficacy in reducing the duration of symptoms and influenza‐related complications in otherwise healthy adults compared to placebo [17]. Due to its effectiveness, baloxavir has been a valuable addition to the options available for influenza management. It received its first global approval in Japan in February 2018 for treating Influenza A and B virus infections [18] and was subsequently licensed in Hong Kong in 2019 [19].

However, baloxavir has not yet been included in the Hong Kong Hospital Authority (HA) Drug Formulary, which standardizes drug policy and drug utilization in all public hospitals and clinics. The primary reason for not including baloxavir into Drug Formulary is the availability of alternatives like oseltamivir. Considering the resistance to oseltamivir, we expect that baloxavir would be a suitable medication to be included, although baloxavir is at a price disadvantage. Several cost‐effectiveness studies conducted in the United States and Japan have evaluated the use of baloxavir compared to other antiviral treatments, particularly laninamivir and oseltamivir, across various populations and settings. These studies consistently indicated that baloxavir is a cost‐effective option for treating influenza in otherwise healthy (OwH) adults or high‐risk (HR) populations [20, 21, 22]. Evidence‐based strategies to promote the uptake of baloxavir and oseltamivir can have significant public health benefits beyond clinical effects, enhancing their cost‐effectiveness. There is an urgent need for a study to fill this evidence in Hong Kong. Building on this foundation, this study evaluates the cost‐effectiveness of baloxavir compared to oseltamivir for treating outpatients in Hong Kong.

## 2 Methods

### 2.1 Study Design

In Hong Kong, the current standard of care primarily involves symptomatic treatment; however, antiviral agents are prescribed by a doctor when indicated [23]. We evaluated the introduction of two antivirals for outpatients presenting within 48 h of symptom onset and with a positive rapid influenza test. Given that symptomatic treatment does not substantially impact the course of the illness or reduce the risk of complications [24], we proposed an alternative baseline as the standard of care.

Influenza risk factors can impact an adult's risk of experiencing severe influenza outcomes from the infection [25]. High‐risk (HR) patients are individuals with conditions identified as risk factors by the CDC [26]. Therefore, the cost‐effective results for the general population were derived from the combined outcomes of the OwH and HR population subsets.

In this analysis, we developed a decision tree to model the progression of influenza and predict the associated costs and quality‐adjusted life‐years (QALYs) for baloxavir, oseltamivir, and baseline 14 days based on the CAPTONE clinical data at the individual‐patient level. The model does not incorporate influenza transmission dynamics or the duration of the influenza season. Discount rates were not applied due to the 14‐days' time horizon. Our study was reported in accordance with the recommendation of the Consolidated Health Economic Evaluation Reporting Standards (CHEERS) statement [27].

### 2.2 Model Structure

**FIGURE 1:**
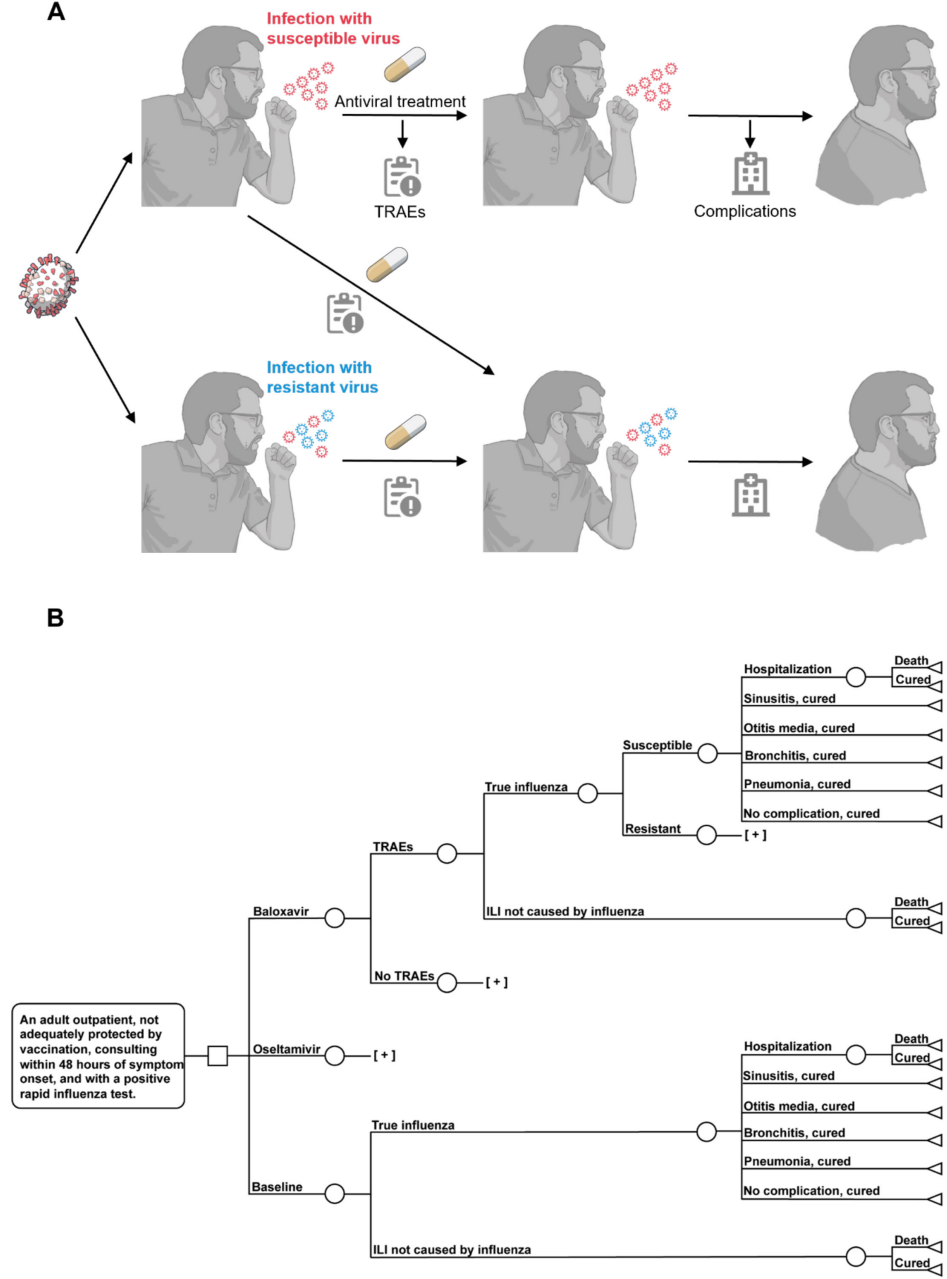
Schematic diagram of the decision‐tree model. Panel (A) outlines potential pathways for the eventual recovery of a patient diagnosed with influenza infection. The sequence begins with the patient taking medication within 48 h of diagnosis. Subsequently, the patient experienced an adverse reaction to the accompanying medication. There are two possible processes through which resistance to drugs. The first possibility is that the infectious viruses are already resistant. The second possibility is that the viruses develop drug resistance during the treatment phase. Both scenarios result in complications due to the ineffectiveness of the treatment. These complications led to the patient's hospitalization, but ultimately, the patient made a full recovery. Panel (B) shows the whole possible path of events in the decision‐tree model. The symbol [+] is used to streamline the schematic representation by eliminating repeated sections. Pathways shown above [+] are applied at the corresponding points. Illustration from NIAID NIH BIOART Source (bioart.niaid.nih.gov/bioart/187, bioart.niaid.nih.gov/bioart/407, bioart.niaid.nih.gov/bioart/329, bioart.niaid.nih.gov/bioart/332). Abbreviations: ILI, influenza‐like illness; TRAEs, treatment‐related adverse events.

The decision tree incorporates individual‐patient health state values, as illustrated in Figure 1. It is assumed that the patient has tested positive for influenza prior to receiving antiviral therapy. The following events are considered for a true influenza patient with an antiviral treatment pathway: development of treatment‐related adverse events (TRAEs), resistance to antivirals, occurrence of influenza‐related complications, hospitalization following complications, and death due to complications or other causes. For patients not receiving antiviral treatment, TRAEs and resistance to antiviral events are not included.

Key model parameters are outlined in Table 1 and Table S1, with detailed information provided in the Supporting Information. The model was constructed in a Java tool, Amua (Ward ZJ, Harvard University, United States) [43]. All analyses were performed using R software, version 4.2.1. (R Foundation for Statistical Computing, Vienna, Austria).

**TABLE 1:** Key input parameters and ranges for sensitivity analyses in otherwise healthy patients.

Parameter	Base‐case value	Limits (low and high value)	Distribution	Distribution parameters	References
Clinical inputs					
Probability of true influenza					
True influenza (influenza diagnostic test)	99%	90%–99%	Beta	Alpha = 3.6, beta = 0.04	[28]
Probability of treatment‐related adverse events (TRAEs)					
Baloxavir	4.40%		None		[29]
Oseltamivir	8.40%		None		
Duration of treatment‐related adverse events (days)					
Baloxavir	6.9	4.373–9.427	LogNormal	mu = 6.90, sigma = 1.29	[17]
Oseltamivir	6.9	4.373–9.427	LogNormal	mu = 6.90, sigma = 1.29	
Duration of influenza symptoms (h)					
Baloxavir	53.5	48.0–58.5	Normal	mu = 53.5, sigma = 2.68	[29]
Oseltamivir	53.8	50.2–56.4	Normal	mu = 53.8, sigma = 1.58	
Resistance patient	80.2	72.6–87.1	Normal	mu = 80.2, sigma = 3.70	
Duration of illness (days)					
Duration of illness (ILI caused by other pathogens)	7.7	7.0–8.5	Gamma	Alpha = 404.91, beta = 52.59	[30]
Duration of outpatient complications	9.23	6.15–12.92	Gamma	Alpha = 28.40, beta = 3.07	[31]
Duration of hospitalization	4	3–7	Gamma	Alpha = 15.37, beta = 0.26	[32]
Duration of ICU	3	2–5	Gamma	Alpha = 15.37, beta = 0.2	[33]
Resistance rate					
Baloxavir	0%		None		
Oseltamivir	0%	0%–40% (scenario)	None	Assume	
Complications‐baloxavir					
Hospitalization	0.01%	0.01%–0.01%	Beta	Alpha = 342.28, beta = 2,986,363	Estimated
Sinusitis	0.80%	0.2%–2.3%	Beta	Alpha = 0.93, beta = 114.88	[29]
Otitis media	0.50%	0.1%–1.9%	Beta	Alpha = 0.45, beta = 88.7	
Bronchitis	2.40%	1.1%–4.5%	Beta	Alpha = 3.52, beta = 143	
Pneumonia	0.50%	0.1%–1.9%	Beta	Alpha = 0.45, beta = 88.7	
Complications‐oseltamivir					
Hospitalization	0.03%	0.03%–0.03%	Beta	Alpha = 339.33, beta = 1,110,388	Estimated
Sinusitis	0.00%	0.0%–1.0%	Beta	Alpha = 0.01, beta = 50	[29]
Otitis media	0.30%	0.0%–1.5%	Beta	Alpha = 0.22, beta = 73.87	
Bronchitis	1.60%	0.6%–3.4%	Beta	Alpha = 2.27, beta = 139.38	
Pneumonia	0.00%	0.0%–1.5%	Beta	Alpha = 0.01, beta = 33	
Complications‐placebo					
Hospitalization	0.05%	0.04%–0.05%	Beta	Alpha = 339.27, beta = 721514.6	[34, 35]
Sinusitis	0.90%	0.1%–3.1%	Beta	Alpha = 0.63, beta = 69.02	[29]
Otitis media	0.00%	0.0%–1.6%	Beta	Alpha = 0.01, beta = 30	
Bronchitis	3.50%	1.5%–6.7%	Beta	Alpha = 3.15, beta = 86.96	
Pneumonia	0.40%	0.0%–2.4%	Beta	Alpha = 3.14, beta = 75.34	
Influenza‐related hospitalizations					
ICU admission rate ^	2.77%	1.97%–3.46%	Beta	Alpha = 25.7, beta = 903.61	[34, 35]
Mortality after influenza‐related hospitalization—baloxavir	0.64%	0.49%–0.74%	Beta	Alpha = 47.6, beta = 7384.16	Estimated
Mortality after influenza‐related hospitalization—oseltamivir	1.21%	0.92%–1.39%	Beta	Alpha = 47.32, beta = 3868.34	
Mortality after influenza‐related hospitalization—placebo ^	1.70%	1.30%–1.96%	Beta	Alpha = 47.09, beta = 2719.31	
Mortality noninfluenza ILI episode	0.10%		None		[31]
Utility inputs					
Disutility‐influenza–related complications					
Hospitalization	0.52	0.42–0.54	Beta	Alpha = 47.42, beta = 43.77	[36]
Hospitalization‐ICU	0.67	0.57–0.69	Beta	Alpha = 54.05, beta = 26.62	[37]
Influenza symptoms *	0.19	0.15–0.23	Beta	Alpha = 34.91, beta = 148.84	[38]
Sinusitis *	0.15	0.12–0.18	Beta	Alpha = 40.67, beta = 230.44	[37]
Otitis media *	0.15	0.12–0.18	Beta	Alpha = 40.67, beta = 230.44	
Bronchitis *	0.15	0.12–0.18	Beta	Alpha = 40.67, beta = 230.44	
Pneumonia *	0.25	0.2–0.3	Beta	Alpha = 1.53, beta = 4.58	
Noninfluenza illness *	0.27	0.22–0.33	Beta	Alpha = 33.24, beta = 89.88	
TRAEs *	0.2	0.16–0.24	Beta	Alpha = 38.21, beta = 152.86	[31]
Cost inputs					
Private clinics/pharmacies (US$)					
Outpatient consultation	195	102.7–287.3	Normal	mu = 195, sigma = 47.0925	[39]
Test	58.5	52–65	Normal	mu = 58.5, sigma = 3.3163	[40]
Baloxavir (Xofluza), per course *	66.3	53.04–79.56	Normal	mu = 66.3, sigma = 6.7654	[41, 42]
Oseltamivir (Tamiflu), per course *	58.5	46.8–70.2	Normal	mu = 58.5, sigma = 5.9695	
Baseline	1.95	1.3–2.6	Normal	mu = 1.95, sigma = 0.3315	[39]
Inpatient	663	530.4–795.6	Normal	mu = 663, sigma = 67.6546	
Intensive care wards	1995.5	1596.4–2394.6	Normal	mu = 1995.5, sigma = 47.099	

*Note:* ^ −10%/+10% change from base‐case tested. * −20%/+20% change from base‐case tested.

### 2.3 Clinical Inputs

The disparity in clinical data between the OwH and the HR is significant. Consequently, the primary clinical data were sourced from two Phase III clinical trials comparing baloxavir with oseltamivir and placebo [44, 45].

Antiviral medications used to treat influenza can lead to specific adverse events, and patients taking these drugs may experience TRAEs. However, we consider the overall incidence of TRAEs rather than adverse events alone. The probabilities of influenza‐related complications were derived from Phase III clinical trials [44, 45]. Hospital/ICU admission rates and mortality probabilities were adapted to the Hong Kong context by first estimating absolute baseline event rates in Hong Kong [34, 35] and then applying the relative treatment effects of baloxavir and oseltamivir [46, 47, 48, 49], expressed as odds ratios or risk ratios in the simulation.

### 2.4 Utility Inputs

For economic evaluations of health interventions, the US Panel on Cost‐Effectiveness in Health and Medicine recommends QALYs as the preferred measure of efficacy [50]. To determine the QALY value, the utility associated with a given health state is multiplied by the number of years spent in that state. Utilities are represented on a scale from 0 to 1, where 0 corresponds to *Death* and 1 represents *Perfect health* [51].

We utilized QALYs lost to compare different treatment strategies. The QALYs lost for each patient were calculated by multiplying the time spent in each health state, weighted by its disutility value. The individual QALY loss (∆Q) was calculated as follows:

$$∆Q=\left(∆u*d\right)/365,$$

where ∆u is the utility loss experienced by the patient and d is the duration in days of the state.

Utility norms were adjusted to account for the TRAEs, the duration of influenza symptoms, and complications caused by influenza, using data from Health Technology Assessments reported by Tappenden et al. [31] and Burch et al. [37]. Due to the limited data, the duration of respiratory complication reductions was applied to all complications. The median time to symptom alleviation for antivirals was obtained from the Phase III clinical trial [44].

### 2.5 Costs Inputs

Costs were evaluated from the public healthcare‐payer's perspective. We assumed that private charges are equivalent to the costs incurred by the public healthcare provider, as represented by the Government of Hong Kong.

The cost analysis focused on direct medical costs, including outpatient consultation, influenza diagnostic tests, medication, hospitalization care, and ICU care (Estimated 1 HKD = 0.13 USD, as of July 12, 2024). The cost per general outpatient clinic visit and per hospital day, encompassing general medical wards and intensive care wards, was estimated using the private charges listed by the HA [39], as well as test and medication costs from local media sources [40, 52].

### 2.6 Scenario Analysis

The primary health‐economic outcome assessed was the incremental cost‐effectiveness ratio (ICER), calculated by dividing the incremental cost of antivirals by the corresponding health outcomes, yielding the additional cost per unit of health benefit [53]. The ICER was evaluated against the willingness‐to‐pay (WTP) threshold to determine cost‐effectiveness. According to the World Health Organization, an ICER of less than one unit of gross domestic product (GDP) per capita is considered highly cost‐effective, and an ICER between one and three units of GDP per capita was considered cost‐effective. Based on Hong Kong's 2023 GDP of USD 50,889 per capita [54], the WTP threshold was set at USD 152,667 per QALY.

The base‐case analysis considered a 0% resistance rate. However, in February 2024, a 40% reduction in susceptibility to oseltamivir was reported among Influenza A (H1N1) viruses [13]. A scenario analysis was conducted to evaluate the impact of antiviral resistance on cost‐effectiveness estimates, alongside an assessment of the effects of changes in the cost of antiviral drugs.

### 2.7 Sensitivity Analysis

Sensitivity analysis was conducted by varying model parameters to identify critical assumptions and evaluate how changes in utilities or probabilities impact decision outcomes.

A deterministic sensitivity analysis (DSA) was conducted to assess the effect of varying individual input parameters on the ICER. One‐way sensitivity analyses were performed on all model parameters associated with uncertainty. Input values were successively replaced with the limits of their 95% confidence intervals or other values representing the plausible ranges of variation. The results were visualized using tornado charts.

A probabilistic sensitivity analysis was performed to evaluate the simultaneous variation of all parameters. We employed Monte Carlo simulation, iterating the model 2000 times with parameter values sampled from their specified distributions, as detailed in Table 1. Probabilistic results were presented on incremental cost‐effectiveness planes and cost‐effectiveness acceptability curves.

## 3 Results

### 3.1 Base‐Case Result

The results of base‐case analysis for baloxavir and oseltamivir are summarized in Table 2. Total payer expenditures were estimated at US$322.74 and US$319.92 per patient for baloxavir and oseltamivir, respectively. In the OwH group, costs were US$320.21 for baloxavir and US$312.96 for oseltamivir, while in the HR group, costs were US$329.32 for baloxavir and US$332.08 for oseltamivir. Notable differences were observed in the cost input details per section, specified inpatient cost, and drug cost across both the OwH and HR subgroups. Baloxavir's ability to avoid high hospitalization costs relative to oseltamivir and baseline partially offset its higher acquisition cost.

**TABLE 2:** Base‐case analysis of treatment strategies from the healthcare‐payer's perspective.

General population				
**Treatment strategy**	**Costs (US$)**			**Total cost (US$)**
	**Outpatient**	**Inpatient**	**Drug cost (per course)**	
Baloxavir	253.50	2.94	66.3	322.74
Oseltamivir	253.50	7.92	58.5	319.92
Baseline	253.50	9.78	1.95	265.23
**Treatment strategy**	**QALYs lost due to**			**Total QALYs lost**
	**TRAEs**	**Influenza symptoms**	**Complications/noninflu illness**	
Baloxavir	0.0001789	0.0012660	0.0002330	0.0016780
Oseltamivir	0.0003123	0.0013171	0.0002549	0.0018843
Baseline	0	0.0018536	0.0004638	0.0023174
**Treatment strategy**	**Incremental costs (US$)** **Baseline strategy: Baseline**	**Incremental QALYs** **Baseline strategy: Baseline**	**ICER (US$)**	**WTP (US$)**
Baloxavir	57.50	0.0006395	89,920.86	152,667.00
Oseltamivir	54.69	0.0004331	126,277.93	
Baseline	NA	NA	NA	
**Treatment strategy**	**Incremental costs (US$)** **Baseline strategy: Oseltamivir**	**Incremental QALYs** **Baseline strategy: Oseltamivir**	**ICER (US$)**	**WTP (US$)**
Baloxavir	2.81	0.0002064	13,625.98	152,667.00
Oseltamivir	NA	NA	NA	

OwH patients				
**Treatment strategy**	**Costs (US$)**			**Total cost (US$)**
	**Outpatient**	**Inpatient**	**Drug cost (per course)**	
Baloxavir	253.50	0.41	66.3	320.21
Oseltamivir	253.50	0.96	58.5	312.96
Baseline	253.50	1.44	1.95	256.89
**Treatment strategy**	**QALYs lost due to**			**Total QALYs lost**
	**TRAEs**	**Influenza symptoms**	**Complications/noninfluenza illness**	
Baloxavir	0.0001664	0.0011488	0.0002378	0.0015523
Oseltamivir	0.0003176	0.0011552	0.0001428	0.0016156
Baseline	0	0.0017221	0.0002666	0.0019887
**Treatment strategy**	**Incremental costs (US$)** **Baseline strategy: baseline**	**Incremental QALYs** **Baseline strategy: baseline**	**ICER (US$)**	**WTP (US$)**
Baloxavir	63.32	0.0004357	145,316.3	152,667.00
Oseltamivir	56.07	0.0003731	150,267.2	
Baseline	NA	NA	NA	
**Treatment strategy**	**Incremental Costs (US$)** **Baseline strategy: Oseltamivir**	**Incremental QALYs** **Baseline strategy: Oseltamivir**	**ICER (US$)**	**WTP (US$)**
Baloxavir	7.25	0.0000626	115,810.6	152,667.00
Oseltamivir	NA	NA	NA	

**HR patients**				
**Treatment strategy**	**Costs (US$)**			**Total cost (US$)**
	**Outpatient**	**Inpatient**	**Drug cost (per course)**	
Baloxavir	253.50	9.521	66.3	329.32
Oseltamivir	253.50	26.08	58.5	338.08
Baseline	253.50	31.54	1.95	286.99
**Treatment strategy**	**QALYs lost due to**			**Total QALYs Lost**
	**TRAEs**	**Influenza symptoms**	**Complications/noninfluenza illness**	
Baloxavir	0.0002117	0.0015718	0.0002204	0.0020040
Oseltamivir	0.0002987	0.0017393	0.0005472	0.0025852
Baseline	0	0.0021966	0.0009780	0.0031747
**Treatment strategy**	**Incremental costs (US$)** **Baseline strategy: Baseline**	**Incremental QALYs** **Baseline strategy: Baseline**	**ICER (US$)**	**WTP (US$)**
Baloxavir	42.34	0.00117073	36,162.19	152,667.00
Oseltamivir	51.10	0.00058946	86,684.27	
Baseline	NA	NA	NA	
**Treatment strategy**	**Incremental costs (US$)** **Baseline strategy: Oseltamivir**	**Incremental QALYs** **Baseline strategy: Oseltamivir**	**ICER (US$)**	**WTP (US$)**
Baloxavir	−8.76	0.0005813	−15,072.12	152,667.00
Oseltamivir	NA	NA	NA	

Both medications demonstrated improvements in QALY, reflecting enhanced quality of life and overall health. Baloxavir resulted in a greater QALY gain compared to oseltamivir (*Δ* = 0.0002064 QALY per patient overall, *Δ* = 0.0000626 QALY in the OwH group, and *Δ* = 0.0005813 QALY in the HR group), attributed to fewer TRAEs and complications, and a shorter duration of symptoms.

Over 14 days, the base‐case ICER of baloxavir versus baseline was US$89,920.86 per QALY. The ICER of baloxavir versus oseltamivir was US$13,625.98 per QALY. In the OwH group, the ICER was US$145,316.3 per QALY for baloxavir and US$150,267.2 per QALY for oseltamivir. Among HR patients, the ICER was US$36,162.19 per QALY for baloxavir and US$86,684.27 per QALY for oseltamivir, with baloxavir being cost‐effective and dominant in this subgroup. Both treatments are cost‐effective at the WTP threshold; however, baloxavir demonstrated greater cost‐effectiveness than oseltamivir under the base‐case assumptions, particularly in HR patients.

### 3.2 Scenario Analysis

Table 3 summarizes the scenario analysis on resistance rates, baloxavir costs, and oseltamivir costs. The impact on QALYs and costs is influenced by changes in the oseltamivir resistance rate. The analysis indicates that as resistance rates increase, QALYs decrease, while costs rise.

**TABLE 3:** Scenario case analysis of treatment strategies from the healthcare‐payer's perspective.

Group	Scenario	Treatment strategy	QALYs lost	Costs (US$)	ICER treatment vs. baseline (US$/QALY)	ICER baloxavir vs. oseltamivir (US$/QALY)
General population	Base case	Baloxavir	0.0016780	322.74	89,920.86	13,625.98
		Oseltamivir	0.0018843	319.92	126,277.93	NA
		Baseline	0.0023174	265.23	NA	NA
	Change resistance against oseltamivir	10%—Oseltamivir	0.0019589	320.11	153,049.21	9348.02
		20%—Oseltamivir	0.0020334	320.30	193,872.95	6864.14
		30%—Oseltamivir	0.0021080	320.48	263,750.76	5241.42
		40%—Oseltamivir	0.0021825	320.67	410,833.55	4098.16
		50%—Oseltamivir	0.0022570	320.85	920,998.65	3249.23
	Change oseltamivir cost	$0—Oseltamivir	0.0025852	279.58	−12,559.17	10,122.49
		$10—Oseltamivir	0.0025852	289.58	4405.52	7776.82
		$20—Oseltamivir	0.0025852	299.58	21,370.21	5431.15
		$30—Oseltamivir	0.0025852	309.58	38,334.9	3085.48
		$40—Oseltamivir	0.0025852	319.58	55,299.59	739.81
		$50—Oseltamivir	0.0025852	329.58	72,264.29	−1605.86
	Change baloxavir cost	$0—Baloxavir	0.0020039	263.02	−20,469.39	475,817.42
		$10—Baloxavir	0.0020039	273.02	−11,927.67	392,197.97
		$20—Baloxavir	0.0020039	283.02	−3385.96	308,578.53
		$30—Baloxavir	0.0020039	293.02	5155.76	224,959.08
		$40—Baloxavir	0.0020039	303.02	13,697.48	141,339.63
		$50—Baloxavir	0.0020039	313.02	22,239.19	57,720.19
OwH patients	Base case	Baloxavir	0.001553	320.21	145,316.3	115,810.6
		Oseltamivir	0.0016156	312.96	150,267.2	NA
		Baseline	0.0019887	256.89	NA	NA
	Change resistance against oseltamivir	10%—Oseltamivir	0.0016846	313.01	184,562.64	54,695.87
		20%—Oseltamivir	0.0017537	313.06	239,020.2	35,635.96
		30%—Oseltamivir	0.0018228	313.1	338,821.88	26,334.29
		40%—Oseltamivir	0.0018918	313.15	580,997.18	20,824.25
		50%—Oseltamivir	0.0019609	313.2	2,028,118.79	17,179.99
	Change oseltamivir cost	$0—Oseltamivir	0.0016156	279.58	−6522.68	20,751.14
		$10—Oseltamivir	0.0016156	289.58	20,279.01	17,595.09
		$20—Oseltamivir	0.0016156	299.58	47,080.69	14,439.05
		$30—Oseltamivir	0.0016156	309.58	73,882.38	11,283.01
		$40—Oseltamivir	0.0016156	319.58	100,684.07	8126.96
		$50—Oseltamivir	0.0016156	329.58	127,485.76	4970.92

	Change baloxavir cost	$0—Baloxavir	0.001553	253.91	−6846.71	−943,191.24
		$10—Baloxavir	0.001553	263.91	16,103.97	−783,462.38
		$20—Baloxavir	0.001553	273.91	39,054.64	−623,733.52
		$30—Baloxavir	0.001553	283.91	62,005.32	−464,004.66
		$40—Baloxavir	0.001553	293.91	84,956	−304,275.79
$50—Baloxavir		0.001553	303.91	107,906.67	−144,546.93
HR patients	Base case	Baloxavir	0.0020039	329.32	36,162.19	−15,072.12
		Oseltamivir	0.0025852	338.08	86,684.27	NA
		Baseline	0.0031747	286.99	NA	NA
	Change resistance against oseltamivir	10%—Oseltamivir	0.002674	338.63	103,148.12	−13,888.49
		20%—Oseltamivir	0.0027628	339.17	126,711.31	−12,981.91
		30%—Oseltamivir	0.0028516	339.72	163,227.97	−12,265.27
		40%—Oseltamivir	0.0029404	340.26	227,428.41	−11,684.54
		50%—Oseltamivir	0.0030292	340.81	370,011.54	−11,204.4
	Change oseltamivir cost	$0—Oseltamivir	0.0025852	279.58	−12559.17	85,570.39
		$10—Oseltamivir	0.0025852	289.58	4405.52	68,366.55
		$20—Oseltamivir	0.0025852	299.58	21,370.21	51,162.71
		$30—Oseltamivir	0.0025852	309.58	38,334.9	33,958.87
		$50—Oseltamivir	0.0025852	319.58	55,299.59	16,755.03
		$50—Oseltamivir	0.0025852	329.58	72,264.29	−448.81
	Change baloxavir cost	$0—Baloxavir	0.0020039	263.02	−20,469.39	−129,133.61
		$10—Baloxavir	0.0020039	273.02	−11,927.67	−111,929.77
		$20—Baloxavir	0.0020039	283.02	−3385.96	−94,725.93
		$30—Baloxavir	0.0020039	293.02	5155.76	−77,522.09
		$40—Baloxavir	0.0020039	303.02	13,697.48	−60,318.25
		$50—Baloxavir	0.0020039	313.02	22,239.19	−43,114.41

Drug cost variations were found to have no effect on QALYs. Critical price thresholds for cost‐effectiveness comparisons between baloxavir and oseltamivir were identified: If all other factors remain constant, oseltamivir becomes the more cost‐effective option when its price drops below US$30. Conversely, baloxavir remains the economical choice if the cost of baloxavir falls below US$95. For OwH patients, the corresponding thresholds are US$56.19 for oseltamivir and US$68.61 for baloxavir, while for HR patients, the thresholds are US$‐39 for oseltamivir and US$163.81 for baloxavir.

### 3.3 Sensitivity Analysis

**FIGURE 2:**
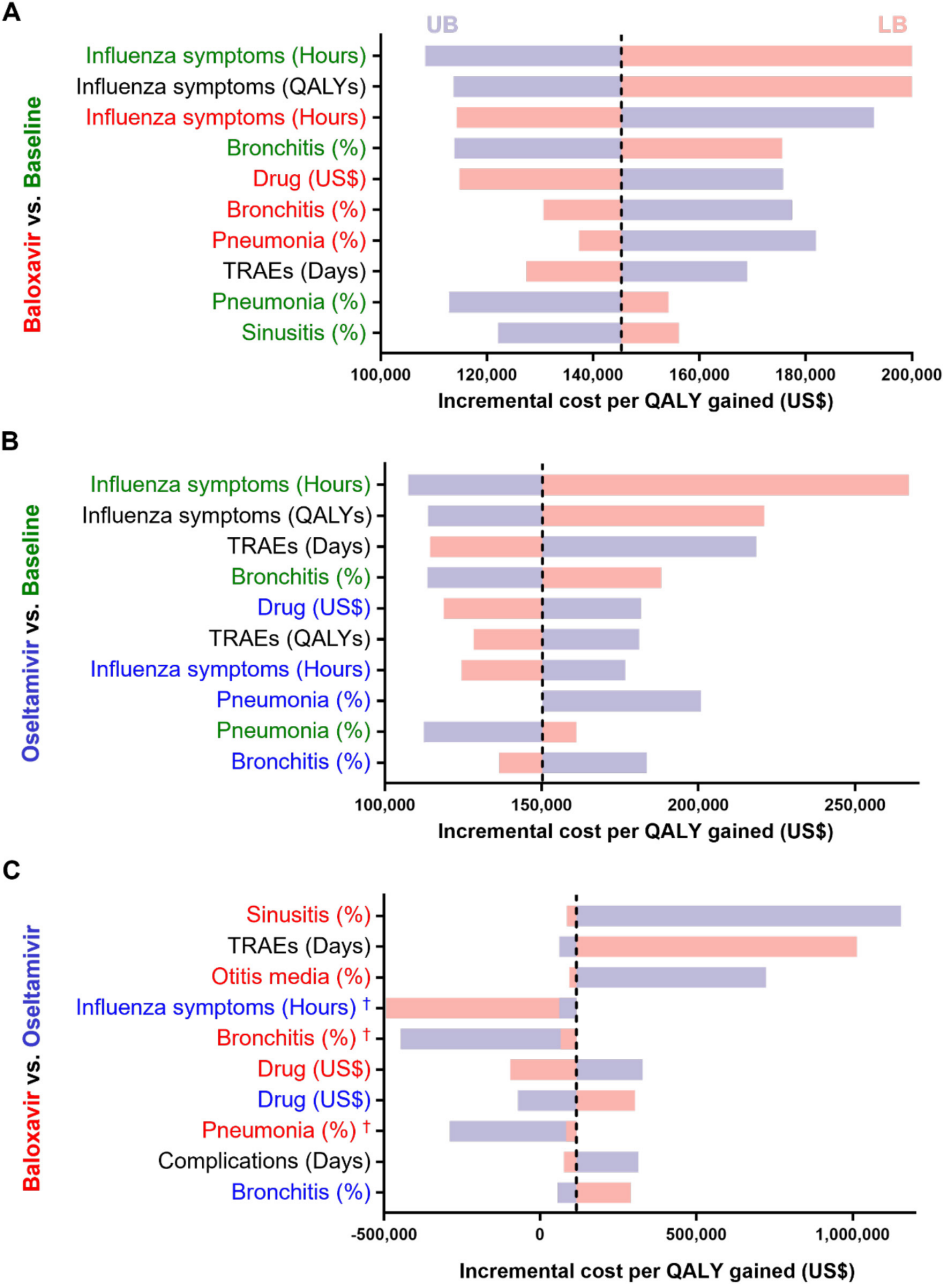
Tornado diagram of deterministic sensitivity analysis for OwH patients. This figure presents a tornado diagram where horizontal bars represent input parameters. The length of each bar reflects the variation in model output when the corresponding parameter is varied within its uncertainty range. Bars are ordered from most to least influential, with the most impactful parameter at the top. The dashed line indicates the base‐case ICER for each strategy. Panels (A), (B), and (C) display the DSA results for Baloxavir versus Baseline, Oseltamivir versus Baseline, and Baloxavir versus Oseltamivir, respectively. Subheading colors correspond to parameters specific to each strategy. When the result of parameter ^†^ is negative, it indicates that the strategy is dominated. Abbreviations: DSA, deterministic sensitivity analysis; ICER, incremental cost‐effectiveness ratio; LB, lower bound; OwH, otherwise healthy; UB, upper bound.

**FIGURE 3:**
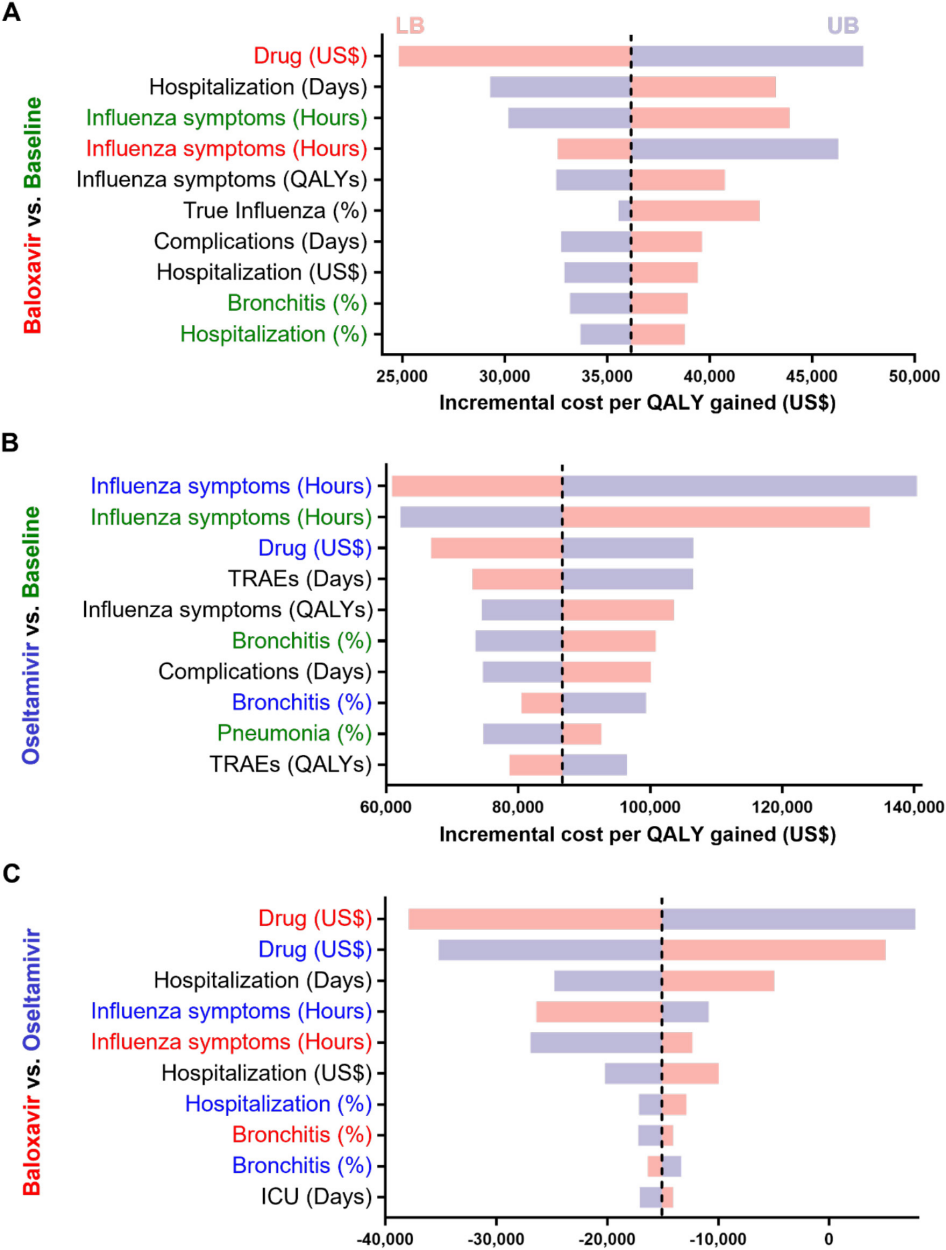
Tornado diagram of deterministic sensitivity analysis for HR patients. This figure presents a tornado diagram where horizontal bars represent the input parameters. The length of each bar reflects the variation in model output when the corresponding parameter is adjusted with its uncertainty range. Bars are ordered from most influential to least influential, with the most impactful parameter at the top. The dashed line indicates the base‐case ICER for each strategy. Panels (A), (B), and (C) display the DSA results for Baloxavir versus Baseline, Oseltamivir versus Baseline, and Baloxavir versus Oseltamivir, respectively. Subheading colors correspond to parameters specific to each strategy. Abbreviations: DSA, deterministic sensitivity analysis; HR, high‐risk; ICER, incremental cost‐effectiveness ratio; LB, lower bound; UB, upper bound.

Figure 2 and Figure 3 (A) display the 10 most influential factors affecting baloxavir treatment relative to baseline. Panels (B) and (C) illustrate results for oseltamivir versus baseline and baloxavir versus oseltamivir, respectively. For the OwH patients, the key factors in the baloxavir versus baseline analysis are the duration of influenza symptoms without medication, QALYs lost due to influenza symptoms, and the duration of influenza symptoms with baloxavir. In contrast, the oseltamivir versus baseline results highlighted the duration of influenza symptoms without medication, QALYs lost due to influenza symptoms, and the duration of TRAEs as the primary factors. In the HR subpopulation, the cost of drugs is the most influential factor across models. The duration of influenza symptoms with medication is the leading factor in the DSA for both medications, followed by the duration of hospitalization without medication. Reductions in the length of hospital stay without medication resulted in decreased costs and increased QALYs, raising the ICER relative to baseline.

**FIGURE 4:**
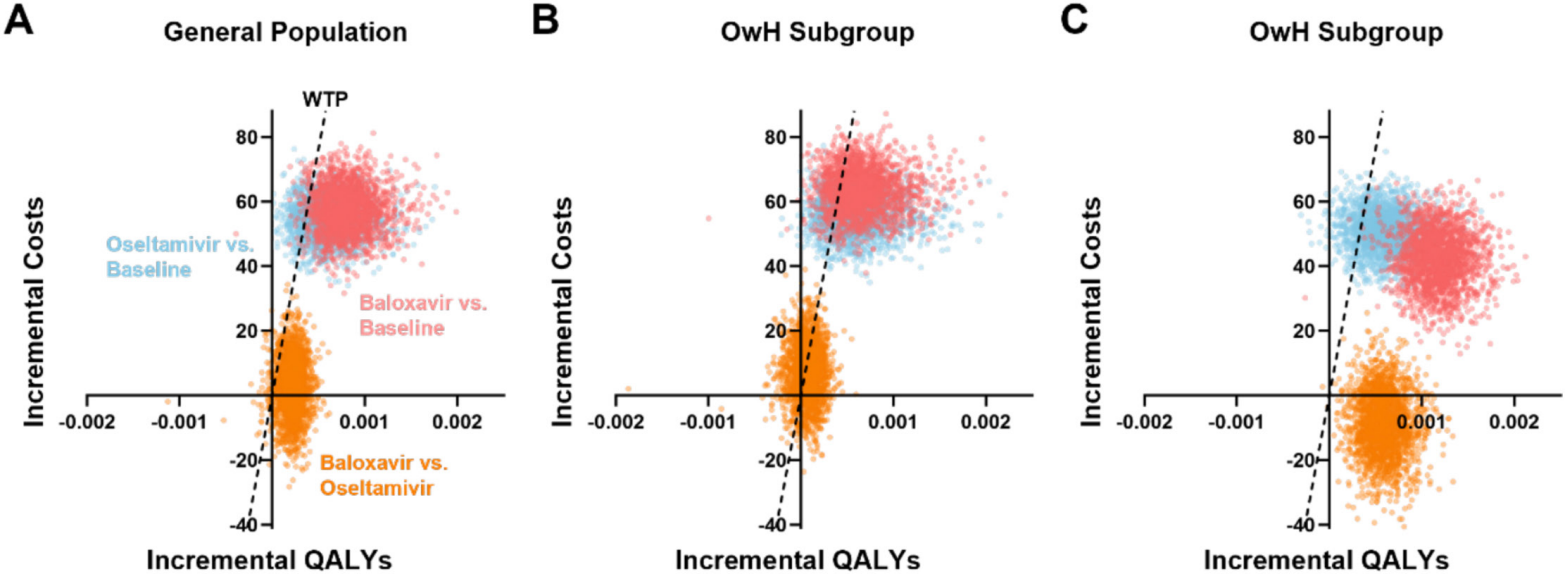
Incremental cost‐effectiveness scatterplot of probabilistic sensitivity analysis. Each dot represents the outcome of a single model simulation, with input values sampled from the statistical distribution of model parameters. The scatter plot illustrates incremental costs and effectiveness of treatments compared to the baseline, as well as between baloxavir and oseltamivir assessments. The dashed line represents the WTP threshold with a slope of US$152,667 per QALY. Panel (A) depicts results for the general population, while Panels (B) and (C) illustrate the results for the OwH and HR populations, respectively. Abbreviations: HR, high‐risk; OwH, otherwise healthy; QALY, quality‐adjusted life‐year; WTP, willingness‐to‐pay.

Figure 4 illustrates the results of the probabilistic sensitivity analysis. The cost‐effectiveness scatter plot highlights the incremental costs and effects of treatments compared to baseline, as well as baloxavir compared to oseltamivir. For most iterations, strategies relative to baseline fall in the first quadrant, indicating improved quality of life with higher costs. In some cases, baloxavir versus oseltamivir falls in the fourth quadrant, signifying increasing QALYs and decreasing costs, indicating the dominance of baloxavir. This trend is consistent across the general and OwH populations, but more pronounced among HR patients, where more iterations fall below the WTP threshold, indicating greater cost‐effectiveness for HR patients. Baloxavir exhibits a superior economic profile over oseltamivir, as evidenced by superior efficacy and inferior expenditure, particularly in HR populations.

**FIGURE 5:**
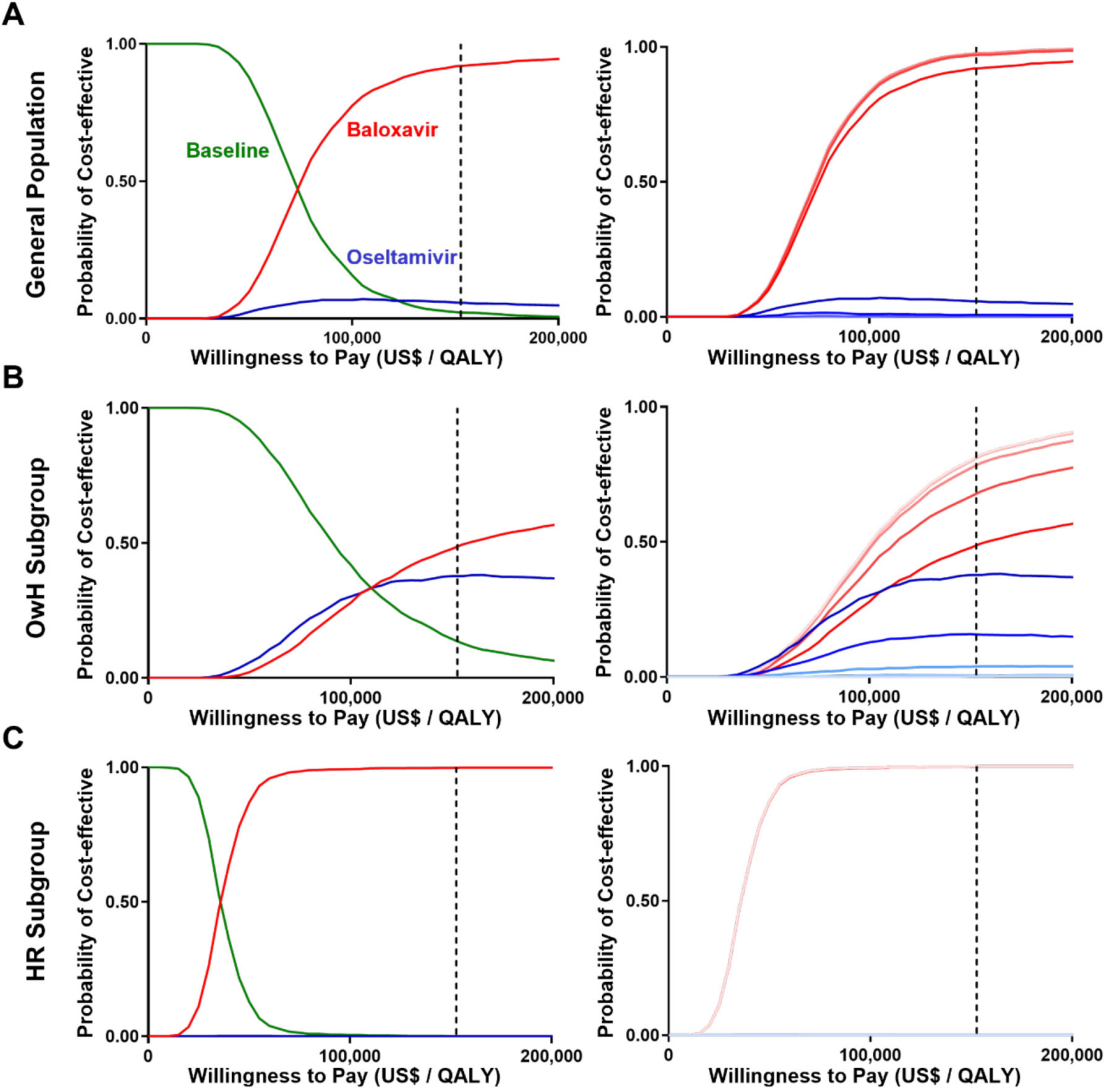
Incremental cost‐effectiveness acceptability curve. The horizontal axis represents the WTP threshold, while the vertical axis indicates the probability that Baloxavir or Oseltamivir is cost‐effective compared to the baseline across varying WTP levels. The dashed vertical line marks the WTP threshold ($152,667 per QALY), based on Hong Kong's GDP per capita. Panels (A) and (C) display the cost‐effectiveness acceptability curves for the basic scenario in the OwH and HR populations, respectively. Panels (B) and (D) illustrate the OwH and HR populations under varying oseltamivir susceptibility scenarios, ranging from 100% to 60%. Lighter colors represent decreasing susceptibility to oseltamivir. Abbreviations: GDP, gross domestic product; HR, high‐risk; OwH, otherwise healthy; QALY, quality‐adjusted life‐year; WTP, willingness‐to‐pay.

The incremental cost‐effectiveness acceptability curve (Figure 5) illustrates the probability of treatments being cost‐effective across various WTP thresholds. At the baseline WTP threshold of $152,667/QALY (Figure 5A), baloxavir is cost‐effective in 92% of simulations, compared to 5.5% for oseltamivir. Oseltamivir has a higher probability of acceptance than baseline at the WTP threshold. In scenarios considering drug resistance, as resistance to oseltamivir increases, the probability of baloxavir offering economic advantages increases. In the OwH subpopulation, baloxavir is cost‐effective in 48.5% of scenarios, compared to 37.5% for oseltamivir with the same WTP level. Oseltamivir is more cost‐effective than baloxavir at lower WTP thresholds but becomes inferior above approximately US$110,000. In the HR population (Figure 5C), baloxavir is cost‐effective in 99% of the scenarios at the baseline WTP threshold, with its probability increasing slightly as oseltamivir susceptibility decreases.

## 4 Discussion

Neuraminidase inhibitors, such as oseltamivir and zanamivir, are the primary antiviral treatments recommended globally for influenza [55]. The introduction of baloxavir marks a significant advancement in antiviral therapy, offering a novel approach to influenza management, though its impact on public health remains uncertain. In Hong Kong, the healthcare system has adapted these global recommendations to address specific local challenges, such as high population density and rapid viral transmission. Although baloxavir has not yet been approved in the HA Drug Formulary, it is highly feasible in the Hong Kong setting. Our assessment, focused on the principal consideration of cost‐effectiveness, contributes to the inclusion of baloxavir in the HA Drug Formulary. Specifically, we used decision model‐based analysis to evaluate the cost‐effectiveness of baloxavir compared to oseltamivir within Hong Kong's unique context, which is marked by high influenza prevalence and significant strain on healthcare services.

We find baloxavir and oseltamivir are cost‐effective for treating influenza in both the OwH and HR populations, under a cost‐effectiveness threshold of US$152,667 per QALY. The base‐case findings align with previous studies, suggesting that baloxavir is likely cost‐effective compared to no treatment or current standard of care in settings such as the United States and Netherlands [20, 56]. Notably, the probability of cost‐effectiveness for baloxavir is significantly higher in the HR group (99%) compared to the OwH group (49%), reflecting greater economic benefits in high‐risk populations. Supporting these findings, Japanese cost‐effectiveness analysis reports that baloxavir has a 64% probability of being cost‐effective in the OwH population and a 72% probability in the HR population compared to laninamivir, another neuraminidase inhibitor [21, 22]. These results highlight the differing economic advantages and acceptance rates of baloxavir across different risk profiles. Our findings strongly support that HR patients are able to prioritize the use of baloxavir as therapy during the flu season in Hong Kong, as recommended by the United States Centers for Disease Control and Prevention [57].

We estimate the ICER of baloxavir versus oseltamivir is US$13,625.98/QALY, well below Hong Kong's per capita GDP, qualifying it as highly cost‐effective and potentially enhancing economic productivity through improved health outcomes [58]. The primary outcomes highlight a significant increase in QALYs, driven by baloxavir's lower probability of TRAEs, faster symptom relief, and reduced incidence of influenza‐related complications. Despite its higher initial cost, baloxavir offers substantial long‐term savings by shortening the duration of symptoms, preventing complications, and reducing hospitalizations. Our findings provide robust evidence supporting the cost‐effectiveness of using baloxavir during seasonal influenza. Similar conclusions have been reported in studies from Japan, the United States, and Europe, where baloxavir demonstrated superior cost‐effectiveness compared to oseltamivir across most modeled scenarios [20, 59, 60]. Although the government has sufficient oseltamivir in stock for 2023, given the short shelf life of the oral suspension and the longer term seasonal influenza activity in 2023–2024, we suggest that the government consider purchasing baloxavir to fill the gap in preparation for the upcoming winter influenza season.

Furthermore, research has shown that baloxavir provides a cost of US$6813 per QALY gain compared to oseltamivir and an ICER of US$669 per QALY gain compared to no treatment from a total population perspective in the United States [20]. Our analysis estimated the ICER for this strategy to be US$13,626 when compared to oseltamivir and US$89,921 when compared to no treatment. A study also showed the ICER for baloxavir was negative, indicating that baloxavir is a dominant strategy [61]. Overall, we observed greater variability in the cost per QALY among basic case scenarios compared to previous studies in other countries. Consequently, this highlights the importance of region‐specific cost‐effectiveness analysis.

The primary strength of our study comes from the scenarios of resistance to antivirals. As known, resistance can develop rapidly during antiviral treatment, potentially compromising therapeutic efficacy [62]. While resistance to oseltamivir is well‐documented, data specific to Hong Kong remain limited. We find the resistance level against medication significantly impacts the cost‐effectiveness of the treatment. In a scenario where oseltamivir resistance rises from 0% to 40% (same as February 2024), the ICER of baloxavir compare with oseltamivir decreases significantly from US$13,626 to US$3249 (Table 3), about one‐fourth of what it would have been in the absence of resistance. Our results indicate that the probability of baloxavir being cost‐effective rises from 92% to 97% with increasing oseltamivir resistance (Figure 5), emphasizing the substantial influence of resistance on economic outcomes. Development of resistance to oseltamivir is not a rare event; one study reported that 30% of oseltamivir‐resistant seasonal Influenza A viruses were isolated from children who received oseltamivir therapy [63]. And resistance to nearly all effective antiviral agents, including baloxavir, has been reported [64]. An Influenza A/HIN1 variant with reduced susceptibility to baloxavir was identified in a hospitalized child who had not received the drug [65]. The interpretation of cost‐effectiveness analyses should account for current levels of influenza activity and resistance, reinforcing the importance of monitoring resistance patterns to inform treatment strategies.

The model also has several limitations. First, it focuses solely on the therapeutic effects of antiviral medications, without considering their potential impact on influenza transmission rates. Effective antiviral medications might reduce both symptoms and the contagiousness of infected individuals, thereby influencing overall community health outcomes [60, 66, 67]. We acknowledge that our work concentrates on the progression of infection within a host, from infection to recovery or death, while ignoring between‐host transmission, which might underestimate the economic benefits of the medications. Similarly, we did not model influenza vaccination coverage, vaccine effectiveness, or the emergence of new strains. Changes in these factors can shift the overall incidence and severity of influenza and, consequently, the aggregate economic impact; they could also modify baseline risks of complications, potentially affecting the magnitude of incremental benefits from baloxavir. A more detailed integration of vaccination strategies and strain‐specific dynamics would require a transmission modeling framework and represents an important area for future research. Second, our analysis primarily addresses direct costs associated with pharmacological interventions, including their immediate economic and health benefits. However, our study does not account for broader economic implications such as indirect costs related to lost productivity, long‐term health effects, or the economic burden on healthcare systems. This exclusion may lead to the undervaluation of outcomes. Nevertheless, its impact can be considered minimal compared to other costs, such as hospitalization. A more comprehensive evaluation incorporating these factors would provide a holistic perspective on the economic impact of influenza treatments.

## 5 Conclusion

In conclusion, our study presents a valuable evaluation of the cost‐effectiveness of baloxavir compared to oseltamivir for treating influenza patients in Hong Kong. Baloxavir emerges as a cost‐effective alternative, particularly advantageous due to its efficacy against antiviral‐resistant strains and its single‐dose administration. Our study provides clear evidence for inclusion of baloxavir in the HA Drug Formulary. By integrating baloxavir into treatment protocols, public resources will be used to maximize the effects of healthcare.

## Author Contributions

**Ruohan Chen:** conceptualization, methodology, formal analysis, writing – original draft, writing – review and editing. **Zengyang Shao:** methodology, writing – review and editing. **Kaiming Bi:** writing – review and editing. **Benjamin John Cowling:** writing – review and editing, supervision. **Zhanwei Du:** conceptualization, methodology, funding acquisition, writing – review and editing, supervision.

## Funding

Supported by the Shenzhen‐Hong Kong‐Macau Science and Technology Project (Category C) (Project no: SGDX20230821091559022), the General Research Fund (grant no. 17103122) from the Research Grants Council, the HMRF Research Fellowship Scheme (grant no. 07210147), financed by the Food and Health Bureau of the Government of Hong Kong S.A.R., China, and the Research Grants Council, Government of the Hong Kong S.A.R., China.

## Ethics Statement

The authors have nothing to report.

## Consent

The authors have nothing to report.

## Conflicts of Interest

B.J.C. consults for AstraZeneca, Fosun Pharma, GlaxoSmithKline, Haleon, Moderna, Novavax, Pfizer, Roche, and Sanofi Pasteur. The other authors report no other potential conflicts of interest.

## Clinical Trial

The authors have nothing to report.

## Supporting information


**Table S1:** Key input parameters and ranges for sensitivity analyses in high‐risk patients.

## Data Availability

Data are available within the article or its Supporting Information. And R scripts are available at https://github.com/RuohanCHEN01/Cost‐effectiveness‐Analysis.
