# Supplementary material for: Evaluating the Health Economic Impacts of Baloxavir Marboxil and Oseltamivir for the Treatment of Influenza in Adult Outpatients in Hong Kong: A Cost‐Effectiveness Analysis
Source: Influenza Other Respir Viruses. 2026 Mar 5;20(3):e70243. doi: 10.1111/irv.70243 (PMC12962020; doi:10.1111/irv.70243)
Supplement: Supplementary file 1 — Table S1: Key input parameters and ranges for sensitivity analyses in high‐risk patients. [file IRV-20-e70243-s001.docx]

## Table S1. Key input parameters and ranges for sensitivity analyses in high risk patients.

| **Parameter** | **Base case value** | **Limits**  **(low and high value)** | **Distribution** | **Distribution parameters** | **References** |
| --- | --- | --- | --- | --- | --- |
| **CLINICAL INPUTS** | | | | | |
| **Probability of true influenza** | | | | | |
| True Influenza  (Influenza diagnostic test) | 99% | 90%-99% | Beta | alpha=3.6 , beta=0.04 | ^1^ |
| **Probability of treatment-related adverse events (TRAEs)** | | | | | |
| Baloxavir | 5.60% |  | None |  | ^2^ |
| Oseltamivir | 7.90% |  | None |  |  |
| **Duration of treatment-related adverse events (days)** | | | | | |
| Baloxavir | 6.9 | 4.373-9.427 | LogNormal | mu=6.90, sigma=1.29 | ^3^ |
| Oseltamivir | 6.9 | 4.373-9.427 | LogNormal | mu=6.90, sigma=1.29 |  |
| **Duration of influenza symptoms (h)** | | | | | |
| Baloxavir | 73.2 | 67.2-85.1 | Normal | mu=73.2, sigma=4.57 | ^2^ |
| Oseltamivir | 81 | 69.4-91.5 | Normal | mu=81, sigma=5.64 |  |
| Resistance patient | 102.3 | 92.7-113.1 | Normal | mu=102.3, sigma=5.20 |  |
| **Duration of illness (days)** | | | | | |
| Duration of illness  (ILI caused by other pathogens) | 7.7 | 7.0-8.5 | Gamma | alpha=404.91, beta=52.59 | ^4^ |
| Duration of outpatient complications | 9.23 | 6.15-12.92 | Gamma | alpha=28.40, beta=3.07 | ^5^ |
| Duration of hospitalization | 5 | 3-7 | Gamma | alpha=24.01, beta=0.21 | ^6^ |
| Duration of ICU | 3 | 2-5 | Gamma | alpha=15.37, beta=0.2 | ^7^ |
| **Complications-Baloxavir** | | | | | |
| Hospitalization | 0.27% | 0.26%-0.296% | Beta | alpha=553.42, beta=203706.1 | Estimated |
| Sinusitis | 0.30% | 0.0%-1.4% | Beta | alpha=0.26, beta=87.12 | ^8^ |
| Otitis media | 0.00% | 0.0%-0.9% | Beta | alpha=0.01, beta=53 |  |
| Bronchitis | 1.80% | 0.7%-3.7% | Beta | alpha=2.52, beta=137.36 |  |
| Pneumonia | 0.00% | 0.0%-0.9% | Beta | alpha=0.01, beta=53 |  |
| **Complications-Oseltamivir** | | | | | |
| Hospitalization | 0.72% | 0.68%-0.77% | Beta | alpha=551.36, beta=75740.99 | Estimated |
| Sinusitis | 0.50% | 0.1%-1.8% | Beta | alpha=0.51, beta=101.79 | ^8^ |
| Otitis media | 0.30% | 0.0%-1.4% | Beta | alpha=0.26, beta=87.12 |  |
| Bronchitis | 2.30% | 1.1%-4.3% | Beta | alpha=3.63, beta=154.05 |  |
| Pneumonia | 0.50% | 0.1%-1.8% | Beta | alpha=0.51, beta=101.79 |  |
| **Complications-Placebo** | | | | | |
| Hospitalization | 0.85% | 0.8%-0.9% | Beta | alpha=550.36, beta=64198.35 | ^9,10^ |
| Sinusitis | 2.10% | 0.9%-4.0% | Beta | alpha=3.26, beta=152.13 | ^8^ |
| Otitis media | 0.80% | 0.2%-2.3% | Beta | alpha=0.93, beta=114.88 |  |
| Bronchitis | 6.00% | 3.8%-8.8% | Beta | alpha=10.19, beta=159.67 |  |
| Pneumonia | 0.80% | 0.2%-2.3% | Beta | alpha=0.93, beta=114.88 |  |
| **Influenza-related hospitalizations** | | | | | |
| ICU Admission rate ^ | 1.11% | 0.98%-1.21% | Beta | alpha=162.01, beta=14487.77 | ^9,10^ |
| Mortality after influenza-related hospitalization - Baloxavir | 2.25% | 2.12%-2.34% | Beta | alpha=711.56, beta=30804.16 | Estimated |
| Mortality after influenza-related hospitalization - Oseltamivir | 4.26% | 3.99%-4.42% | Beta | alpha=696.78, beta=15659.5 |  |
| Mortality after influenza-related hospitalization - Placebo ^ | 6.00% | 5.63%-6.22% | Beta | alpha=684.13, beta=10718.07 |  |
| Mortality non-influenza ILI episode | 0.10% |  | None |  | ^5^ |
| **UTILITY INPUTS** | | | | | |
| **Disutility-Influenza related complications** | | | | | |
| Hospitalization | 0.52 | 0.42-0.54 | Beta | alpha=47.42, beta=43.77 | ^11^ |
| Hospitalization-ICU | 0.67 | 0.57-0.69 | Beta | alpha=54.05, beta=26.62 | ^12^ |
| Influenza symptoms * | 0.19 | 0.15-0.23 | Beta | alpha=34.91, beta=148.84 | ^13^ |
| Sinusitis * | 0.15 | 0.12-0.18 | Beta | alpha=40.67, beta=230.44 | ^12^ |
| Otitis media * | 0.15 | 0.12-0.18 | Beta | alpha=40.67, beta=230.44 |  |
| Bronchitis * | 0.15 | 0.12-0.18 | Beta | alpha=40.67, beta=230.44 |  |
| Pneumonia * | 0.25 | 0.2-0.3 | Beta | alpha=1.53, beta=4.58 |  |
| Non-influenza illness * | 0.27 | 0.22-0.33 | Beta | alpha=33.24, beta=89.88 |  |
| TRAEs * | 0.2 | 0.16-0.24 | Beta | alpha=38.21, beta=152.86 | ^5^ |
| **COST INPUTS** |  |  |  |  |  |
| **Private Clinics/Pharmacies (US$)** | | | | | |
| Outpatient consultation | 195 | 102.7-287.3 | Normal | mu=195, sigma=47.0925 | ^14^ |
| Test | 58.5 | 52-65 | Normal | mu=58.5, sigma=3.3163 | ^15^ |
| Baloxavir (Xofluza), per course * | 66.3 | 53.04-79.56 | Normal | mu=66.3, sigma=6.7654 | ^16,17^ |
| Oseltamivir (Tamiflu), per course * | 58.5 | 46.8-70.2 | Normal | mu=58.5, sigma=5.9695 |  |
| Baseline | 1.95 | 1.3-2.6 | Normal | mu=1.95, sigma=0.3315 | ^14^ |
| Inpatient | 663 | 530.4-795.6 | Normal | mu=663, sigma=67.6546 |  |
| Intensive Care Wards | 1995.5 | 1596.4-2394.6 | Normal | mu=1995.5, sigma=47.099 |  |

^ -10%/+10% change from base case tested.

* -20%/+20% change from base case tested.

# Reference

1. INDICAID COVID-19/FLU A&B rapid antigen test. Available at https://phasescientific.com/products-and-services/indicaid/indicaid-covid-flu-rat. Accessed September 2, 2024.

2. Clinicaltrials.gov. Available at https://clinicaltrials.gov/study/NCT02949011?cond=Influenza&term=Baloxavir%20Marboxil&aggFilters=status:com&rank=7&tab=results#outcome-measures. Accessed October 4, 2024.

3. Hayden Frederick G., Sugaya Norio, Hirotsu Nobuo, et al. Baloxavir Marboxil for Uncomplicated Influenza in Adults and Adolescents. N Engl J Med 2018;379:913-923.

4. Butler CC, van der Velden AW, Bongard E, et al. Oseltamivir plus usual care versus usual care for influenza-like illness in primary care: an open-label, pragmatic, randomised controlled trial. Lancet 2020;395:42-52.

5. Tappenden P, Jackson R, Cooper K, et al. Amantadine, oseltamivir and zanamivir for the prophylaxis of influenza (including a review of existing guidance no. 67): a systematic review and economic evaluation. Health Technol Assess 2009;13:iii, ix-xii, 1-246.

6. Su S, Chaves SS, Perez A, et al. Comparing clinical characteristics between hospitalized adults with laboratory-confirmed influenza A and B virus infection. Clin Infect Dis 2014;59:252-255.

7. Shah S, McManus D, Bejou N, et al. Clinical outcomes of baloxavir versus oseltamivir in patients hospitalized with influenza A. J Antimicrob Chemother 2020;75:3015-3022.

8. Miller FP, Vandome AF, McBrewster J, eds. Clinicaltrials.gov. Alphascript Publishing; 2010. 68 p.

9. Wu P, Presanis AM, Bond HS, Lau EHY, Fang VJ, Cowling BJ. A joint analysis of influenza-associated hospitalizations and mortality in Hong Kong, 1998-2013. Sci Rep 2017;7:929.

10. Wong JY, Cheung JK, Presanis AM, et al. Assessing the impact of influenza epidemics in Hong Kong. J Infect Dis March 2025.

11. Lee BY, McGlone SM, Bailey RR, et al. To test or to treat? An analysis of influenza testing and antiviral treatment strategies using economic computer modeling. PLoS One 2010;5:e11284.

12. Burch J, Paulden M, Conti S, et al. Antiviral drugs for the treatment of influenza: a systematic review and economic evaluation. Health Technol Assess 2009;13:1-265, iii-iv.

13. Fragaszy EB, Warren-Gash C, White PJ, et al. Effects of seasonal and pandemic influenza on health-related quality of life, work and school absence in England: Results from the Flu Watch cohort study. Influenza Other Respi Viruses 2018;12:171-182.

14. Hospital Authority : Fees and Charges. Available at https://www.ha.org.hk/visitor/ha_visitor_index.asp?Content_ID=10045&Lang=ENG&Dimension=100&Parent_ID=10044&Ver=HTML. Accessed August 15, 2024.

15. [Flu prevention] Provide list of hospitals and prices for rapid testing clinics. Available at https://www.ohpama.com/207371/%E7%94%9F%E6%B4%BB%E7%86%B1%E8%A9%B1/%E7%94%9F%E6%B4%BB%E7%86%B1%E8%A9%B1/%E9%A0%90%E9%98%B2%E6%B5%81%E6%84%9F-%E6%8F%90%E4%BE%9B%E5%BF%AB%E9%80%9F%E6%B8%AC%E8%A9%A6%E8%A8%BA%E6%89%80%E9%86%AB%E9%99%A2%E5%90%8D%E5%96%AE%E5%8F%8A%E5%83%B9%E9%8C%A2/. Accessed August 15, 2024.

16. EDD Health. Available at https://www.eddmall.com/goods_detail/Tamiflu-Oseltamivir. Accessed December 18, 2025.

17. EDD Health. Available at https://www.eddmall.com/goods_detail/Xofluza-baloxavir-marboxil-40mg. Accessed December 18, 2025.
